# Late Outcome after Surgery for Type-A Aortic Dissection

**DOI:** 10.3390/jcm9092731

**Published:** 2020-08-24

**Authors:** Mikko Jormalainen, Peter Raivio, Fausto Biancari, Caius Mustonen, Hannu-Pekka Honkanen, Maarit Venermo, Antti Vento, Tatu Juvonen

**Affiliations:** 1 Heart and Lung Center, Helsinki University Hospital, 00029 Helsinki, Finland; mikko.jormalainen@hus.fi (M.J.); peter.raivio@hus.fi (P.R.); antti.vento@hus.fi (A.V.); tatu.juvonen@hus.fi (T.J.); 2 Research Unit of Surgery, Anesthesia and Critical Care, University of Oulu, 90014 Oulu, Finland; caius.mustonen@student.oulu.fi (C.M.); hannu-pekka.honkanen@student.oulu.fi (H.-P.H.); 3 Department of Surgery, University of Turku, 20014 Turku, Finland; 4 Department of Vascular Surgery, Helsinki University Hospital, 00029 Helsinki, Finland; maarit.venermo@hus.fi

**Keywords:** aortic dissection, type A aortic dissection, reoperation, reintervention

## Abstract

The aim of this study was to evaluate all-cause mortality and aortic reoperations after surgery for Stanford type A aortic dissection (TAAD). We evaluated the late outcome of patients who underwent surgery for acute TAAD from January 2005 to December 2017 at the Helsinki University Hospital, Finland. We studied 309 patients (DeBakey type I TAAD: 89.3%) who underwent repair of TAAD. Aortic root repair was performed in 94 patients (30.4%), hemiarch repair in 264 patients (85.4%) and partial/total aortic arch repair in 32 patients (10.4%). Hospital mortality was 13.6%. At 10 years, all-cause mortality was 34.9%, and the cumulative incidence of aortic reoperation or late aortic-related death was 15.6%, of any aortic reoperation 14.6%, reoperation on the aortic root 6.6%, on the aortic arch, descending thoracic and/or abdominal aorta 8.7%, on the descending thoracic and/or abdominal aorta 6.4%, and on the abdominal aorta 3.8%. At 10 years, cumulative incidence of reoperation on the distal aorta was higher in patients with a diameter of the descending thoracic aorta ≥35 mm at primary surgery (cumulative incidence in the overall series: 13.2% vs. 4.0%, SHR 3.993, 95%CI 1.316–12.120; DeBakey type I aortic dissection: 13.6% vs. 4.5%, SHR 3.610, 95%CI 1.193–10.913; patients with dissected descending thoracic aorta: 15.8% vs. 5.9%, SHR 3.211, 95%CI 1.067–9.664). In conclusion, surgical repair of TAAD limited to the aortic segments involved by the intimal tear was associated with favorable survival and a low rate of aortic reoperations. However, patients with enlarged descending thoracic aorta at primary surgery had higher risk of late reoperation. Half of the distal aortic reinterventions were performed on the abdominal aorta.

## 1. Introduction

Surgery for acute Stanford Type A aortic dissection (TAAD) is associated with substantial early mortality and morbidity [1,2]. In these patients, surgical strategy is dictated by the extent of TAAD, the site of the intimal tear and the coexistence of malperfusion syndromes. Most surgeons prefer an expeditious surgical repair of the ascending aorta, while a more complex repair of the aortic root and aortic arch/descending thoracic aorta is performed only when necessary [3]. However, more extensive surgical or hybrid aortic repair during primary surgery or as a completion procedure may prevent degeneration of the dissected aorta [4]. These extensive aortic interventions are supposed to protect the fragile remaining aorta from degeneration and to confer a reduction in volume and lead to thrombosis of the false lumen. However, these procedures can be associated with adverse events [5] and do not completely prevent late events [6,7,8,9]: these aspects may not justify their associated increased risks and costs. In this scenario of uncertainty, evaluation of the long-term outcome after surgery for TAAD is of importance to estimate the risk of late aortic-related complications and to identify patients who may most benefit from a more extensive surgical and/or completion endovascular procedure. These issues were investigated in the present institutional series.

## 2. Material and Methods

### 2.1. Study Characteristics

Data on consecutive patients who underwent surgical repair for acute TAAD from January 2005 to December 2017 at the Helsinki University Hospital, Finland, were retrospectively collected. Permission to conduct this study was obtained by the Review Board of our Institution. Data were retrospectively collected into an electronic datasheet with prespecified variables by two coauthors (C.M., H-P.M.) and underwent checking of its completeness and consistency by other three coauthors (M.J, P.R., F.B). Data on the maximal diameter of each segment of the thoracic aorta were retrieved from preoperative computed tomography scans. Clinical variables were defined according to the EuroSCORE II definition criteria [10]. Patient’s risk was stratified according to the EuroSCORE II [10].

Our Institutional policy was to treat only the aortic segments involved by the intimal tear along with hemiarch replacement and to avoid aortic root and/or aortic arch replacement when these segments were not involved by the intimal tear and their diameters were within a normal range. Surgical technique was previously described [11]. In brief, hemiarch replacement was defined as any proximal arch repair beyond the level of the innominate artery but not involving the arch vessels. Partial and total arch replacement was defined as any replacement of supra-aortic vessels as an island or individual branched grafts. After discharge, computed tomography or magnetic resonance controls were scheduled at 3 months, 1 year, 2 years and then every second year after surgery.

### 2.2. Outcomes

The primary outcomes of this study were all-cause mortality and any distal aortic reoperation, i.e., surgical or endovascular procedure on the aortic arch, descending thoracic and/or abdominal aorta. These were the subjects of multivariable regression analyses.

Secondary outcomes were hospital mortality from all causes, stroke or global brain ischemia, amount of red blood cell transfusion, reoperation for bleeding, deep sternal wound infection/mediastinitis, acute kidney injury, renal replacement therapy and length of stay in the intensive care unit as well as aorta-related mortality and any surgical or endovascular repeat procedure on each segment of the aorta, i.e., the aortic root/ascending aorta, aortic arch, descending aorta and/or abdominal aorta. Only repeat procedures which involved surgical resection or endovascular repair of any aortic segment or aortic tube prosthesis qualified as aortic reinterventions.

In this series, fatal events occurring 3 months or later after surgery were considered as late deaths [12]. Reoperations on the aorta were defined as any surgical or endovascular procedure for the treatment of the complication related to aortic dissection, such as the development of a pseudoaneurysm, aneurysm or visceral ischemia. The decision to reoperate on the aorta was based on the presence of an aneurysm or pseudoaneurysm ≥55 mm in diameter or with signs of significant growth, graft infection, aortic native or prosthetic valve stenosis, regurgitation or endocarditis. Hospital mortality was defined as all-cause death occurred during the index hospitalization.

Stroke was defined as any focal (limited to a specific vascular territory of the brain) or global (diffusely involving several areas of the brain) neurological syndrome occurring during the index hospitalization caused by ischemia and/or hemorrhage lasting >24 h. The diagnosis and nature of stroke was based on computed tomography findings and was confirmed by a neurologist. Any infection involving the deep tissues/sternal bone, and/or the mediastinum was defined as deep sternal wound infection/mediastinitis [13]. Acute kidney injury was defined according to the Kidney Disease Improving Global Outcomes (KDIGO) criteria [14].

Data on the date and cause of death were retrieved from the national registry Statistics Finland. The most recent causes of death were classified as unknown when they were not yet given on Statistics Finland. Electronic files were reviewed to identify any aortic reoperation performed in patients residing in the Helsinki catchment area.

### 2.3. Statistical Analysis

Continuous variables are summarized as means and standard deviations and as medians and interquartile ranges. Categorical variables are summarized as counts and percentages. Receiving operating characteristic curve analysis was performed to estimate the area under the curve of the maximal diameter of the aorta in predicting the need of repeat aortic procedure. The Youden’s test was used to estimate the best cutoff of the aortic diameter in predicting aortic reoperations. The Kaplan–Meier method with the log-rank test was used to estimate late all-cause mortality. Independent predictors of late all-cause mortality were identified with the Cox proportional hazards method with the backward stepwise method with a probability of entry of the covariates of 0.05 and or removal of 0.10. Competing risk analysis was performed for late reoperation considering all-cause mortality as a competing event. Predictors of aortic reoperation were identified using the Fine–Gray’s test. Regression analyses were performed including covariates with *p* < 0.05 in univariate analysis for the Cox proportional hazards analysis and *p* < 0.01 for competing risk analysis. Hazard ratios (HR) and subdistribution hazard ratios (SHR) were estimated with their 95% confidence intervals (CI). Late all-cause mortality is summarized as rates and 95%CI and repeat aortic procedures as cumulative incidences and 95%CI. *p* < 0.05 was considered statistically significant. Statistical analyses were performed using Stata v. 15.1 (StataCorp LLC, College Station, TX, USA) and SPSS v. 25.0 (IBM Corporation, New York, NY, USA) statistical software.

## 3. Results

### 3.1. Study Cohort

Three-hundred-and-nine consecutive patients underwent surgical repair for acute TAAD from January 2005 to December 2017 at the Helsinki University Hospital, Finland. The baseline characteristics and operative data of these patients are summarized in Table 1; Table 2. Data on the maximal diameter of each segment of the thoracic aorta were retrieved from preoperative computed tomography scans of 244 (79.0%) patients.

DeBakey type I TAAD was observed in 276 patients (89.3%). Aortic root replacement or repair was performed in 94 patients (30.4%), hemiarch repair in 264 patients (85.4%) and partial or total aortic arch repair in 32 patients (10.4%).

Secondary outcomes are summarized in Table S1. Hospital mortality was 13.6% and 30-day mortality 15.7%.

### 3.2. Late All-Cause Mortality

**Figure 1:**
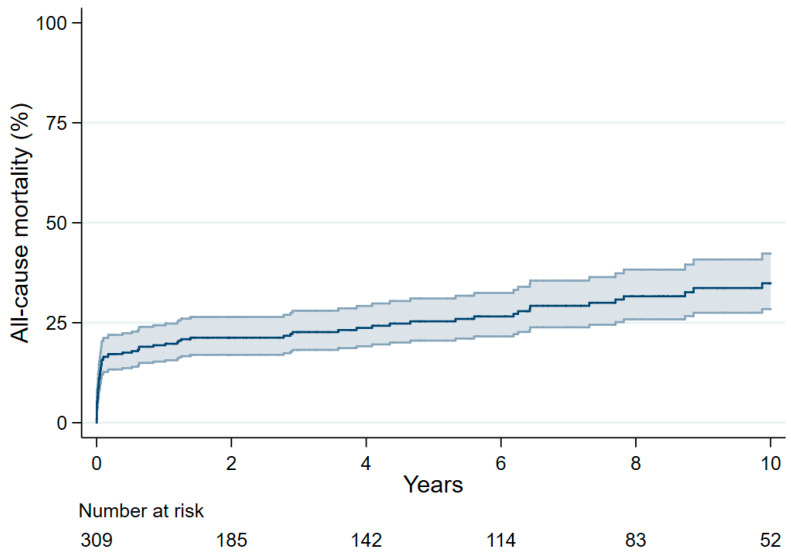
Survival after surgical repair of Stanford type A aortic dissection.

The mean follow-up of this series was 4.7 ± 4.5 years (median 3.3 years, interquartile range 8.2 years). Thirty-nine (12.6%) patients died 3 months or later after surgery and their causes of death are summarized in Table S2. The cause of death of six patients who died recently was not yet available from Statistics Finland. Three patients (7.8% of late deaths) died after rupture of thoracic and/or abdominal aortic aneurysm. Overall, nine patients died of aortic rupture or unknown cause (23.1% of late deaths). All-cause mortality at 1, 3, 5 and 10 years was 19.4%, 22.7%, 25.3% and 34.9%, respectively (Table S3, Figure 1). Among 258 patients who survived at 3-month after primary surgery, 10-year all-cause mortality was 21.4%.

Age, diabetes, resuscitation in the operating room, dissection of the epiaortic vessels and partial/total aortic arch repair were independent predictors of early and late all-cause mortality (Table 1; Table 2). When early adverse events where included in the regression model, age, resuscitation in operating room, dissection of the epiaortic vessels and partial/total aortic arch repair along with postoperative stroke/global brain ischemia and acute kidney injury were associated with increased risk of early and late all-cause mortality (Table 3). Age, preoperative anemia and preoperative estimated glomerular filtration rates were independent predictors of late all-cause mortality among 3-month survivors (Table S4).

### 3.3. Aortic Reoperations

Thirty-three (10.7%) patients required 39 surgical or endovascular procedures for aortic aneurysm, pseudoaneurysm or visceral malperfusion. These procedures and their main indications are summarized in Table S5. The mean interval between primary surgery and reoperation was 4.3 ± 3.9 years (median, 2.9 years, interquartile range 6.0 years). Reoperations on the aortic root were performed in 12 patients (36.4%), the aortic arch in 5 (15.2%), the descending thoracic aorta in 9 (27.3%) and the abdominal aorta in 11 (33.3%). Four patients underwent reoperation with root replacement for isolated aortic native valve or prosthesis related complications. Aortic valve replacement was performed in eight patients, while aortic valve sparing procedure was performed in one patient (Table S5). Overall, 21 patients underwent distal aortic reoperation, 11 patients underwent reoperation on the aortic root, and 1 patient underwent both a proximal and distal aortic reoperation. Reoperation of the abdominal aorta was required in 50.0% of distal aortic reoperations.

At 10 years, the cumulative incidence of aortic reoperation or late aortic-related death was 15.6%, of any aortic reoperation 14.6%, of reoperation on the aortic root 6.6%, on the aortic arch, descending thoracic and/or abdominal aorta 8.7%, on the descending thoracic and/or abdominal aorta 6.4% and on the abdominal aorta 3.8% (Table S3).

Multivariate competing risk analysis showed that peripheral arterial disease, DeBakey type I TAAD, level of distal aortic anastomosis and the diameter of the descending aorta increased the risk of distal aortic reoperation (Table 1 and Table 2).

Among 33 patients who required an aortic reoperation, the mean follow-up after reintervention was 3.5 ± 3.3 years (median 2.9 years, interquartile range 3.8 years). Among these patients, only one patient with ruptured abdominal aortic aneurysm died early after the operation (on the 40th postoperative day). Five-year all-cause mortality in this subset of patients was 25.6%.

### 3.4. Outcome of Patients with DeBakey Type I Aortic Dissection

At 10-year, all-cause mortality of patients with DeBakey type I aortic dissection was 34.9% (95%CI 28.4–42.3). At this interval, the cumulative incidence of aortic reoperation or aortic-related death was 17.1% (95%CI 11.7–23.2), of any aortic reoperation 15.9% (95%CI 10.8–22.0), of reoperation on the aortic root 7.2% (95%CI 3.8–11.9), of reoperation on the aortic arch, descending thoracic and/or abdominal aorta 9.5% (95%CI 5.7–14.6), of reoperation on the descending thoracic and/or abdominal aorta 7.1% (95%CI 3.9–11.4), and of reoperation on the abdominal aorta 4.2% (95%CI 1.9–7.9).

### 3.5. Maximal Diameter of the Aorta and Risk of Aortic Reoperation

Data on the maximal diameter of different segments of the thoracic aorta were retrieved from the preoperative computed tomography scan of 244 patients (Table 1). Competing risk analysis showed that the maximal diameter of the aortic root (*p* = 0.528) was not associated with an increased risk of reoperation on the aortic root. Similarly, the maximal diameter of the aortic root (*p* = 0.837), ascending aorta (*p* = 0.985) and aortic arch (*p* = 0.538) were not associated with an increased risk of repeat distal aortic procedures. Instead, the maximal diameter of the descending thoracic aorta (*p* < 0.0001; area under the ROC curve 0.728, 95%CI 0.611–0.845; adjusted for the aortic segments involved by the dissection, SHR 1.164, 95%CI 1.104–1.129) was an independent predictor of distal aortic procedure (Table 1). When the maximal diameter of the descending thoracic aorta was dichotomized according to a cutoff of 35 mm (174 vs. 70 patients, 22.7%; sensitivity 73%, specificity 66%), a diameter of the descending thoracic aorta ≥35 mm was associated with higher cumulative incidence of reoperation on the distal aorta at 10 years (cumulative incidence in the overall series: 13.2% (95%CI 4.6–26.4) vs. 4.0% (95%CI 1.2–9.4), SHR 3.993, 95%CI 1.316–12.120; DeBakey type I aortic dissection: 13.6% vs. 4.5%, SHR 3.610, 95%CI 1.193–10.913; patients with dissected descending thoracic aorta: 15.8% vs. 5.9%, SHR 3.211, 95%CI 1.067–9.664).

All three patients who died of late aortic rupture had a descending thoracic aorta >40 mm in diameter at primary surgery. At 10 years, the cumulative incidence of rupture of the distal aorta or distal aortic reoperation was higher when the diameter of the descending thoracic aorta was ≥35 mm (overall series: 22.8% vs. 15.0%, SHR 2.109, 95%CI 0.983–4.526; DeBakey type I aortic dissection: 23.6% vs. 16.9%, SHR 1.902, 95%CI 0.887–4.078; patients with dissected descending thoracic aorta: 27.0% vs. 17.1%, SHR 1.923, 95%CI 0.876–4.218), but the difference did not reach statistical significance.

## 4. Discussion

The present study showed that: (1) late survival of patients operated for TAAD was satisfactory and, when a policy of strict postoperative surveillance was applied, only a small number of them died of aortic-related events; (2) primary surgery was limited to the aortic segment involved by the intimal tear with a low risk of aortic reoperation; (3) reoperations on the dissected abdominal aorta accounted for half of the distal aortic procedures; (4) patients with a descending thoracic aorta diameter ≥35 mm at primary surgery had a significantly increased risk of distal aortic reoperations.

This study showed that a strategy to repair only aortic segments involved by the intimal tear of the aortic dissection along with hemiarch replacement is associated with satisfactory early and late survival and a low risk of aortic reoperations. It is worth noting that 70% of patients underwent primary surgery without resection of the aortic root and 10-year cumulative incidence of reintervention on the aortic root was still only 6.6% (Table S3). These findings are in line with other studies sharing the same surgical approach [3,15]. These results should be viewed whilst considering that previous studies did not clearly report on reoperation on the abdominal aorta [3]. Indeed, in the present series, half of patients requiring a distal aortic reoperation underwent surgical or endovascular treatment of the dissected abdominal aorta, most often as an isolated repair.

Although a pooled analysis showed that aortic arch repair in patients with TAAD can be performed without increased risk [3], previous studies [16] and our experience showed that aortic arch surgery was associated with increased mortality (30-day: 25.2% vs. 14.6%; 10-year: 53.3% vs. 33.6%, adjusted HR 2.770, 95%CI 1.562–4.913). Furthermore, most studies demonstrated that aortic arch repair with or without the use of frozen elephant trunk technique seems not to decrease the risk of distal aortic reoperation [6,7,8,9,16,17,18,19,20,21,22], whose freedom rates at 10 years may range from 78.0% to 92.9% [17,21]. We recognize that there are also studies from series including mostly ascending aorta/hemiarch repairs reporting 10-year freedom from reoperation as low as 61% to 78% [15,23], and decreased aortic reoperations after total arch replacement [15,24]. Still, the present findings suggested that a strategy of primary surgical repair limited to the aortic segment involving the intimal tear was associated with a rather low operative mortality without having jeopardized the durability of the aortic repair. In fact, competing risk analysis which considered patient’s death as a competing risk, showed that, in the overall series, the cumulative incidence of reintervention on the aorta was quite low (14.6% at 10 years).

Suzuki et al. [22] and the present analysis showed that the diameter of the descending thoracic aorta was a risk factor for aortic reoperations. Leontyev et al. [24]. demonstrated that the risk of distal aortic reoperation increased significantly when the postoperative descending aortic diameter was greater than 40 mm. These findings suggest that a more extensive repair at primary surgery and/or strict postoperative surveillance should be considered in patients with dilated descending thoracic aorta. Further studies are needed to better define the prognostic impact of enlarged descending thoracic aorta and the potential benefit of extensive primary surgery in these patients.

Finally, the present study confirmed that either proximal or distal aortic reoperations, even if technically demanding, are associated with excellent early and late outcome [25]. In fact, in our series only one patient died 40 days after emergency surgery of ruptured abdominal aortic aneurysm, and 5-year survival in this subset of patients was 74.4%. The excellent results of repeat aortic intervention and the low rate of late aortic rupture in TAAD patients are further arguments in favor of a primary repair limited to the aortic segments involved by the intimal tear, while repeat, extensive surgical and/or hybrid repair should be indicated in those few patients with late aneurysmatic degeneration or complications related to the aortic dissection.

The first major limitation of this study is its retrospective nature. Second, these patients were operated on in the emergency setting, which might prevent complete collection of data on patients’ comorbidities at admission. Third, we do not have complete data on the extent of aortic dissection because a few patients did not undergo preoperative computed tomography due to unstable hemodynamic conditions. In some cases, computed tomography was performed at other institutions and the scan images were not available for review. However, the description of the surgical procedure and the findings of intraoperative transesophageal ultrasound made the classification of the extent of TAAD in patients without preoperative computed tomography possible as well. Fourth, the decision about the extent of primary surgery might have varied between surgeons. Still, in this series, the surgical strategy was rather homogenous as the number of aortic arch procedures was rather small, whilst most patients underwent hemiarch repair. Final, the relatively limited small size of this series and the number of events might introduce bias in the regression analyses.

The rather long follow-up, the availability of data on repeated procedures on patients from our large catchment area as well as the data on all-cause mortality retrieved from the Finnish national statistical institute are the strengths of this study.

## 5. Conclusions

In conclusion, this study showed that surgical repair of TAAD limited to the aortic segments involved by the intimal tear was associated with favorable early and late survival, and a rather low rate of repeat procedures on the aorta. Half of the reinterventions were performed on the abdominal aorta and further studies are needed to evaluate the fate of this segment of the aorta in TAAD patients. Enlarged descending thoracic aorta increased the risk of aortic reoperation, which may indicate more extensive surgical repair and/or strict postoperative surveillance.

## Figures and Tables

**Table 1:** Baseline characteristics and predictors of outcome in patients who underwent surgical repair for type-A aortic dissection. Risk estimates refer to independent predictors of adverse events.

Covariates	Overall Dataset 309 Patients	All-Cause Mortality		Repeat Procedures on the Distal Aorta	
		Univariate Analysis *p*-Value	Multivariate Analysis HR, 95%CI	Univariate Analysis *p*-Value	Multivariate Analysis SHR, 95%CI
Age, mean/median (years)	61.7 (13.2)/63.3 (17.7)	<0.0001	1.056, 1.033–1.079	0.822	
Female	102 (33.0)	0.951		0.380	
Anemia ^a^	129 (41.7)	0.045		0.298	
eGFR, mean/median (mL/min/1.73 m^2^)	79.3 (27.3)/77.6 (33.0)	0.027		0.277	
Peripheral vascular disease	13 (4.2)	0.001		<0.0001	1.14 × 10^−8^, 4.08 × 10^−9^–3.16 × 10^−8^
Diabetes	25 (8.1)	0.031	1.996, 1.048–3.801	0.999	
Stroke	17 (5.5)	0.848		0.775	
Pulmonary disease	22 (7.1)	0.372		0.169	
Coronary artery disease	29 (9.4)	0.025		0.913	
Acute myocardial infarction	32 (10.4)	0.307		0.295	
LVEF ≤50%	57 (19.3)	0.081		0.034	
Previous cardiac surgery	13 (4.2)	0.518		0.982	
Acute neurological event	73 (23.7)	0.054		0.757	
Cardiogenic shock	68 (22.0)	0.002		0.387	
Resuscitation in operating room	7 (2.3)	<0.0001	6.886, 2.451–19.348	-	
Cardiac tamponade	87 (28.3)	0.015		0.634	
Aortic rupture	49 (15.9)	0.062		0.744	
Bicuspid aortic valve	26 (8.4)	0.134		0.523	
Connective tissue disorder	7 (2.2)	0.993		-	
Preop. malperfusion at CT ^b^	138 (46.3)	0.310		0.029	
Cerebral vessel dissection	108 (35.0)	0.150	1.980, 1.249–3.140	0.248	
DeBakey Type II dissection	33 (10.7)	0.463		<0.0001	2.02 × 10^−8^, 8.52 × 10^−9^–4.76 × 10^−8^
Max. aortic diameter (mm) ^c^					
Aortic root, mean/median	44.9 (8.3)/44.0 (11.0)	0.830		0.837	
Ascending aorta, mean/median	51.4 (9.8)/50.0 (10.0)	0.448		0.985	
Aortic arch, mean/median	38.0 (6.1)/37.0 (9.0)	0.392		0.538	
Descending thoracic aorta, mean/median	33.3 (6.0)/33.0 (6.0)	0.004		<0.0001	1.118, 1.064–1.175
EuroSCORE II, mean/median (%)	13.9 (13.3)/9.0 (11.9)	<0.0001		0.295	

Continuous variables are report as mean and standard deviation and median and interquartile range in parentheses; categorical variables are reported as counts and percentages in parentheses; estimated glomerular filtration rate according to the MDRD criteria; LVEF, left ventricular ejection fraction; CT, computed tomography; CI, confidence interval; HR, hazard ratio; SHR, subdistribution hazard ratio. ^a^, hemoglobin <12.0 g/L in female and <13.0 g/L in men; ^b^, computed tomography was not performed preoperatively in 19 patients; ^c^, data available from 244 patients.

**Table 2:** Operative data and predictors of outcome in patients who underwent surgical repair for type-A aortic dissection.

Covariates	Overall Dataset 309 Patients	All-Cause Mortality		Repeat Procedure on the Distal Aorta	
		Univariate Analysis *p*-Value	Multivariate Analysis HR, 95%CI	Univariate Analysis *p*-Value	Multivariate Analysis SHR, 95%CI
Arterial cannulation ^a^		<0.0001		<0.0001	
Direct aortic	80 (25.9)				
Common femoral artery	131 (42.4)				
Axillary/subclavian artery	96 (31.1)				
Left ventricle apex	2 (0.6)				
Aortic root repair/replacement	94 (30.4)	0.618		0.832	
Type of aortic root repair		0.652		<0.0001	
Interposition graft procedure	210 (68.0)				
Interposition graft + AVR	5 (1.6)				
Bentall-DeBono procedure	84 (27.2)				
David procedure	9 (2.9)				
Yacoub procedure	1 (0.3)				
Concomitant major cardiac procedure	43 (13.9)	0.127		0.751	
Open distal anastomosis	289 (93.5)	0.295		0.644	
Distal aortic anastomosis		0.144		<0.0001	
Ascending aorta	277 (89.6)				-
Between innominate a. and left CCA	4 (1.3)				1.06 × 10^−8^, 1.88 × 10^−9^–5.94 × 10^−8^
Between left CCA and left subclavian a.	6 (1.9)				6.029, 1.883–19.306
Descending thoracic aorta	22 (7.1)				0.674, 0.128–3.522
Hemiarch repair	264 (85.4)	0.098		0.373	
Partial or total arch repair	32 (10.4)	0.025	2.770, 1.562–4.913	0.738	
Partial arch repair	10 (3.2)				
Total arch repair	22 (7.1)				
Frozen elephant trunk procedure	4 (1.3)	0.028		-	
Aortic cross clamp time, mean/median (min)	103 (39)/92 (47)	0.143		0.553	
Cardiopulmonary bypass time mean/median (min)	198 (62)/191 (76)	0.313		0.680	
Hypothermic circulatory arrest	273 (88.3)	0.920		0.694	
Hypothermic circulatory arrest time mean/median (min)	27 (11)/26 (35)	0.260		0.172	
Antegrade cerebral perfusion	57 (18.5)	0.009		0.445	
Antegrade cerebral perfusion time mean/median (min)	39 (25)/32 (35)	0.898		0.336	
Lowest core temperature, mean/median (°C)	19 (2)/18 (1)	0.821		0.850	

Continuous variables are reported as mean and standard deviation and median and interquartile range in parentheses; categorical variables are reported as counts and percentages in parentheses; ^a^, some patients had double arterial cannulation sites; aortic valve replacement; CABG, coronary artery bypass grafting; CCA, common carotid artery; CI, confidence interval; HR, hazard ratio; SHR, subdistribution hazard ratio.

**Table 3:** Independent preoperative, operative and postoperative risk factors associated with poor late all-cause mortality after surgery for type A aortic dissection.

Outcomes	Multivariate Analysis HR, 95%CI
Age	1.066, 1.040–1.092
Resuscitation in operating room	3.912, 1.176–13.021
Partial or total arch repair	2.493, 1.291–4.811
Cerebral vessel dissection	1.653, 1.025–2.666
Postop. stroke/global brain ischemia	1.989, 1.197–3.305
Postop. acute kidney injury	2.156, 1.297–3.582

## Supplementary Materials

The following are available online at https://www.mdpi.com/2077-0383/9/9/2731/s1, Table S1: Early adverse events in patients who underwent surgical repair for type-A aortic dissection, Table S2: Main causes of death in patients who died three months or later after surgery for type A aortic dissection, Table S3: Estimates of late outcomes of patients who underwent surgical repair for type-A aortic dissection, Table S4: Baseline and operative factors associated with poor late survival among 3-month survivors after surgery for type A aortic dissection Table S5: Repeat procedures on the aorta after surgery for type A aortic dissection.

## References

[1] Pape L.A., Awais M., Woznicki E.M., Suzuki T., Trimarchi S., Evangelista A., Myrmel T., Larsen M., Harris K.M., Greason K. (2015). Presentation, diagnosis, and outcomes of acute aortic dissection: 17-year trends from the International Registry of Acute Aortic Dissection. J. Am. Coll. Cardiol..

[2] Kohl L.P., Isselbacher E.M., Majahalme N., Evangelista A., Russo M.J., Hutchison S., Bossone E., Suzuki T., Pyeritz R.E., Gleason T.G. (2018). Comparison of outcomes in DeBakey Type A I versus A II aortic dissection. Am. J. Cardiol..

[3] Pan E., Gudbjartsson T., Ahlsson A., Fuglsang S., Geirsson A., Hansson E.C., Hjortdal V., Jeppsson A., Järvelä K., Mennander A. (2018). Low rate of reoperations after acute type A aortic dissection repair from The Nordic Consortium Registry. J. Thorac. Cardiovasc. Surg..

[4] Hsu H.L., Chen Y.Y., Huang C.Y., Huang J.H., Chen J.S. (2016). The Provisional Extension to Induce Complete Attachment (PETTICOAT) technique to promote distal aortic remodelling in repair of acute DeBakey type I aortic dissection: Preliminary results. Eur. J. Cardiothorac. Surg..

[5] Hsu C.P., Huang C.Y., Wu F.Y. (2019). Relationship between the extent of aortic replacement and stent graft for acute DeBakey type I aortic dissection and outcomes: Results from a medical center in Taiwan. PLoS ONE.

[6] Shrestha M., Haverich A., Martens A. (2017). Total aortic arch replacement with the frozen elephant trunk procedure in acute DeBakey type I aortic dissections. Eur. J. Cardiothorac. Surg..

[7] Smith H.N., Boodhwani M., Ouzounian M., Saczkowski R., Gregory A.J., Herget E.J., Appoo J.J. (2017). Classification and outcomes of extended arch repair for acute Type A aortic dissection: A systematic review and meta-analysis. Interact. Cardiovasc. Thorac. Surg..

[8] Poon S.S., Theologou T., Harrington D., Kuduvalli M., Oo A., Field M. (2016). Hemiarch versus total aortic arch replacement in acute type A dissection: A systematic review and meta-analysis. Ann. Cardiothorac. Surg..

[9] Kreibich M., Berger T., Rylski B., Chen Z., Beyersdorf F., Siepe M., Czerny M. (2020). Aortic reinterventions after the frozen elephant trunk procedure. J. Thorac. Cardiovasc. Surg..

[10] Nashef S.A., Roques F., Sharples L.D., Nilsson J., Smith C., Goldstone A.R., Lockowandt U. (2012). EuroSCORE II. Eur. J. Cardiothorac. Surg..

[11] Jormalainen M., Raivio P., Mustonen C., Honkanen H.P., Vento A., Biancari F., Juvonen T. Direct aortic versus peripheral arterial cannulation in surgery for type-A aortic dissection. Ann. Thorac. Surg..

[12] Hirji S., McGurk S., Kiehm S., Ejiofor J., Ramirez-Del Val F., Kolkailah A.A., Berry N., Sobieszczyk P., Pelletier M., Shah P. (2019). Utility of 90-day mortality vs 30-day mortality as a quality metric for transcatheter and surgical aortic valve replacement outcomes. JAMA Cardiol..

[13] Mangram A.J., Horan T.C., Pearson M.L., Silver L.C., Jarvis W.R. (1999). Guideline for prevention of surgical site infection, 1999. Hospital Infection Control Practices Advisory Committee. Infect. Control. Hosp. Epidemiol..

[14] (2012). Acute Kidney Injury Work Group: Kidney Disease: Improving Global Outcomes (KDIGO) Clinical practice guideline for acute kidney injury. Kidney Int..

[15] Shiono M., Hata M., Sezai A., Niino T., Yagi S., Negishi N. (2006). Validity of a limited ascending and hemiarch replacement for acute type A aortic dissection. Ann. Thorac. Surg..

[16] Ohtsubo S., Itoh T., Takarabe K., Rikitake K., Furukawa K., Suda H., Okazaki Y. (2002). Surgical results of hemiarch replacement for acute type A dissection. Ann. Thorac. Surg..

[17] Ma W.G., Chen Y., Zhang W., Li Q., Li J.R., Zheng J., Liu Y.M., Zhu J.M., Sun L.Z. (2020). Limited versus extended repair for acute type A aortic dissection: Long-term outcomes of the Beijing approach beyond 10 years. J. Cardiovasc. Surg. (Torino).

[18] Heo W., Song S.W., Kim T.H., Lee J.S., Yoo K.J., Cho B.K., Lee H.S. (2019). Differential impact of intimal tear location on aortic dilation and reintervention in acute type I aortic dissection after total arch replacement. J. Thorac. Cardiovasc. Surg..

[19] Zhang L., Yu C., Yang X., Sun X., Qiu J., Jiang W., Wang D. (2019). Hybrid and frozen elephant trunk for total arch replacement in DeBakey type I dissection. J. Thorac. Cardiovasc. Surg..

[20] Roselli E.E., Idrees J.J., Bakaeen F.G., Tong M.Z., Soltesz E.G., Mick S., Johnston D.R., Eagleton M.J., Menon V., Svensson L.G. (2018). Evolution of simplified frozen elephant trunk repair for acute DeBakey type I dissection: Midterm outcomes. Ann. Thorac. Surg..

[21] Inoue Y., Matsuda H., Omura A., Seike Y., Uehara K., Sasaki H., Kobayashi J. (2018). Long-term outcomes of total arch replacement with the non-frozen elephant trunk technique for Stanford Type A acute aortic dissection. Interact. Cardiovasc. Thorac. Surg..

[22] Suzuki T., Asai T., Kinoshita T. (2018). Predictors for late reoperation after surgical repair of acute type A aortic dissection. Ann. Thorac. Surg..

[23] Dohle D.S., El Beyrouti H., Brendel L., Pfeiffer P., El-Mehsen M., Vahl C.F. (2019). Survival and reinterventions after isolated proximal aortic repair in acute type A aortic dissection. Interact. Cardiovasc. Thorac. Surg..

[24] Leontyev S., Haag F., Davierwala P.M., Lehmkuhl L., Borger M.A., Etz C.D., Misfeld M., Gutberlet M., Mohr F.W. (2017). Postoperative changes in the distal residual aorta after surgery for acute type A aortic dissection: Impact of false lumen patency and size of descending aorta. Thorac. Cardiovasc. Surg..

[25] Rylski B., Beyersdorf F., Kari F.A., Schlosser J., Blanke P., Siepe M. (2014). Acute type A aortic dissection extending beyond ascending aorta: Limited or extensive distal repair. J. Thorac. Cardiovasc. Surg..

