# Supplementary material for: Late Outcome after Surgery for Type-A Aortic Dissection
Source: J Clin Med. 2020 Aug 24;9(9):2731. doi: 10.3390/jcm9092731 (PMC7563246; doi:10.3390/jcm9092731)
Supplement: Supplementary file 1 [file jcm-09-02731-s001.pdf]

SUPPLEMENTARY MATERIALS

Supplementary Table 1: Early adverse events in patients who underwent surgical repair for type-A aortic dissection.

Outcomes	Overall Dataset 309 patients
Hospital death	42 (13.6)
30-day death	47 (15.2)
Stroke/global brain ischemia	71 (23.0)
De novo stroke/global brain ischemia[a]	36 (15.3)
Red blood cell transfusion, mean/median (units)	3.3 (4.1)/4.1 (5.0)
Reoperation for bleeding	40 (12.9)
Postop. IABP or ECMO	4 (1.3)
DSWI or mediastinitis	14 (4.5)
Acute kidney injury	137 (47.9)
Renal replacement therapy	42 (13.6)
Intensive care unit stay, mean/median (days)	6.2 (6.3)/4.0 (6.0)

Continuous variables are report as mean and standard deviation and median and interquartile range in parentheses; categorical variables are reported as counts and percentages in parentheses; IABP, intra-aortic balloon pump; ECMO, extracorporeal membrane oxygenation; DSWI, deep sternal wound infection; [a], excluding patients with preoperative acute neurological event.

Supplementary Table 2: Main causes of death in patients who died three months or later after surgery for type A aortic dissection.

Main cause of late death	No.
Cancer	7
Unknown	6
Sepsis	4
Myocardial infarction	3
Pneumonia	3
Ruptured of the dissected thoracic and/or abdominal aortic aneurysm	3
Alzheimer’s disease	2
Heart failure	2
Intra-cranial hemorrhage	2
Neurodegenerative disease	2
Stroke	2
Drug intoxication	1
Renal failure	1
Trauma	1
Total	39

Supplementary Table 3: Estimates of late outcomes of patients who underwent surgical repair for type-A aortic dissection.

Interval	Mortality	Reoperation on the Aorta or Aortic-Related Death	Any Reoperation on the Aorta	Reoperation on the Aortic Root	Reoperation on the Aortic Arch, Descending Thoracic and/or Abdominal Aorta	Reoperation on the Descending Thoracic and/or Abdominal Aorta	Reoperation on the Abdominal Aorta
1 year	19.4 (15.3–24.4)	2.2 (0.9–4.4)	2.2 (0.9–4.4)	0.4 (0.3–1.9)	1.8 (0.7–0.4)	1.5 (0.5–3.5)	0.8 (0.2–2.5)
2 years	21.3 (17.0–26.5)	4.8 (2.7–7.8)	4.8 (2.7–7.8)	1.1 (0.3–3.0)	3.7 (1.9–6.5)	3.4 (1.7–6.1)	1.9 (0.7–4-1)
3 years	22.7 (18.2–28.0)	6.6 (4.0–10.0)	6.6 (4.0–10.0)	1.5 (0.5–3.6)	5.1 (2.8–8.2)	4.3 (2.3–7.3)	2.4 (1.0–4.8)
4 years	23.7 (19.1–29.2)	7.6 (4.7–11.3)	7.1 (4.3–10.6)	1.5 (0.5–3.6)	5.6 (3.2–8.9)	4.3 (2.3–7.3)	2.4 (1.0–4.8)
5 years	25.3 (20.5–31.1)	9.7 (6.3–13.9)	8.7 (5.5–12.7)	3.2 (1.4–6.1)	6.1 (3.5–9.6)	4.3 (2.3–7.3)	2.4 (1.0–4.8)
6 years	26.6 (21.6–32.5)	9.7 (6.3–13.9)	8.7 (5.5–12.7)	3.2 (1.4–6.1)	6.1 (3.5–9.6)	4.9 (2.6–8.2)	3.0 (1.3–5.9)
7 years	29.2 (23.8–35.5)	12.3 (8.3–17.2)	11.2 (7.4–16.0)	5.8 (3.0–9.9)	6.1 (3.5–9.6)	4.9 (2.6–8.2)	3.0 (1.3–5.9)
8 years	31.6 (25.8–38.3)	14.5 (10.0–20.0)	13.5 (9.1–18.8)	6.6 (0.4–11.0)	7.6 (4.5–11.7)	6.4 (3.6–10.4)	3.8 (1.1–7.2)
9 years	33.7 (27.5–40.8)	14.5 (10.0–20.0)	13.5 (9.1–18.8)	6.6 (0.4–11.0)	7.6 (4.5–11.7)	6.4 (3.6–10.4)	3.8 (1.1–7.2)
10 years	34.9 (28.4–42.3)	15.6 (10.7–21.4)	14.6 (9.8–20.2)	6.6 (0.4–11.0)	8.7 (5.1–13.4)	6.4 (3.6–10.4)	3.8 (1.1–7.2)

Values are rates and cumulative incidences with 95% confidence intervals in parentheses.

Supplementary Table 4: Baseline and operative factors associated with poor late survival among 3-month survivors after surgery for type A aortic dissection.

Outcomes	Multivariate Analysis HR, 95%CI
Age	1.045, 1.004–1.087
Anemia	2.585, 1.284–5.205
Estimated glomerular filtration rate	0.987, 0.975–1.000

Supplementary Table 5: Repeat procedures on the aorta after surgery for type A aortic dissection.

Patient No.	Time to Repeat Procedure (Years)	Repeat Procedures on the Aorta	Aortic Segment Treated
1	2.9	Surgical repair of thoracoabdominal aortic aneurysm; Endovascular repair of aneurysm of the descending thoracic aorta	Thoracic descending aorta; Abdominal aorta
2	0.1	Surgical repair of the aortic arch for cerebral vessel malperfusion	Aortic arch
3	1.0	Surgical repair of the abdominal aorta for visceral ischemia	Abdominal aorta
4	6.1	Surgical repair of pseudoaneurysm of the right coronary button anastomosis; Surgical repair of pseudoaneurysm; Repeat Bentall-DeBono procedure	Aortic root
5	4.6	Surgical repair of pseudoaneurysm of the proximal aortic anastomosis	Aortic root
6	0.2	Surgical repair of pseudoaneurysm of the proximal aortic anastomosis	Aortic root
7	0.9	Surgical repair of mycotic abdominal aortic aneurysm	Abdominal aorta
8	9.4	Surgical repair of aortic arch; endovascular repair of pseudoaneurysm of the aortic arch	Aortic arch
9	2.5	Surgical repair of aortic arch aneurysm	Aortic arch
10	3.2	Surgical repair of aortic arch and descending thoracic aortic aneurysm with frozen elephant trunk; Surgical repair of abdominal aortic aneurysm; Endovascular repair of aneurysm of the descending thoracic aorta	Aortic arch; Thoracic descending aorta; Abdominal aorta
11	2.2	Surgical repair of aneurysm of the descending thoracic aorta	Thoracic descending aorta
12	1.5	Surgical repair of aneurysm of the descending thoracic aorta	Thoracic descending aorta

13	10.4	Surgical repair of aneurysm of the descending thoracic aorta	Thoracic descending aorta
14	1.3	Surgical repair of abdominal aortic aneurysm	Abdominal aorta
15	1.1	Surgical repair of abdominal aortic aneurysm	Abdominal aorta
16	14.0	Surgical repair of abdominal aortic aneurysm	Abdominal aorta
17	0.8	Surgical repair of abdominal aortic aneurysm	Abdominal aorta
18	12.3	Surgical repair of abdominal aortic aneurysm	Abdominal aorta
19	11.1	Surgical repair of abdominal aortic aneurysm	Abdominal aorta
20	7.4	Endovascular repair of juxtarenal abdominal aortic aneurysm	Abdominal aorta
21	7.3	Endovascular repair of aneurysm of the descending thoracic aorta	Thoracic descending aorta
22	0.1	Endovascular repair of aneurysm of the descending thoracic aorta	Thoracic descending aorta
23	1.4	Endovascular repair of aneurysm of the descending thoracic aorta	Thoracic descending aorta
24	0.3	Endovascular repair of aneurysm of the descending thoracic aorta	Thoracic descending aorta
25	6.1	David procedure for enlargement of the aortic root	Aortic root
26	4.1	Bentall-DeBono procedure for aortic valve regurgitation	Aortic root; Aortic valve
27	6.7	Bentall-DeBono procedure for aortic valve endocarditis	Aortic root; Aortic valve
28	6.9	Bentall-DeBono procedure for aortic valve endocarditis	Aortic root; Aortic valve
29	1.0	Bentall-DeBono procedure for pseudoaneurysm of the aortic root	Aortic root

30	7.8	Bentall-DeBono procedure for aortic prosthetic valve endocarditis and pseudoaneurysm of the proximal aortic anastomosis	Aortic root
31	4.1	Bentall-DeBono procedure and aortic aorch repair for pseudoaneurysm of the proximal aortic anastomosis, aortic arch aneurysm and prosthesis valve stenosis	Aortic root; Aortic arch, Aortic valve prosthesis
32	1.1	Aortic valve replacement for aortic valve regurgitation and repair of tear of the non-coronary sinus	Aortic root; Aortic valve
33	2.0	Aortic valve replacement for aortic valve regurgitation and repair for pseudoaneurysm of the proximal aortic anastomosis	Aortic root; Aortic valve
